# First-Principles Study of Silicon–Tin Alloys as a High-Temperature Thermoelectric Material

**DOI:** 10.3390/ma15124107

**Published:** 2022-06-09

**Authors:** Shan Huang, Suiting Ning, Rui Xiong

**Affiliations:** Key Laboratory of Artificial Micro- and Nano-Structures of Ministry of Education, School of Physics and Technology, Wuhan University, Wuhan 430072, China; shanhuang@whu.edu.cn (S.H.); stning@whu.edu.cn (S.N.)

**Keywords:** thermoelectrics, silicon–tin alloy, density functional theory, electron–phonon coupling

## Abstract

Silicon–germanium (SiGe) alloys have sparked a great deal of attention due to their exceptional high-temperature thermoelectric properties. Significant effort has been expended in the quest for high-temperature thermoelectric materials. Combining density functional theory and electron–phonon coupling theory, it was discovered that silicon–tin (SiSn) alloys have remarkable high-temperature thermoelectric performance. SiSn alloys have a figure of merit above 2.0 at 800 K, resulting from their high conduction band convergence and low lattice thermal conductivity. Further evaluations reveal that Si_0.75_Sn_0.25_ is the best choice for developing the optimum ratio as a thermoelectric material. These findings will provide a basis for further studies on SiSn alloys as a potential new class of high-performance thermoelectric materials.

## 1. Introduction

Thermal energy may be readily converted into electric energy using thermoelectric materials, without the need for complex devices. The figure of merit (*ZT*) is a thermoelectric material criterion that is expressed as

(1) $$ZT=\frac{S^{2}\sigma }{\kappa }T$$

where S is the Seebeck coefficient, σ is the electrical conductivity, κ is the total thermal conductivity, and $S^{2}\sigma$ is the power factor (PF). Thermoelectric materials, such as SiGe [1,2], Bi_2_Te_3_ [3,4,5], and SnSe [6,7,8,9], have been extensively explored since they have been demonstrated to exhibit good electron and thermal transport properties. As is widely known, SiGe, as a traditional high-temperature thermoelectric material, is extensively investigated. However, owing to its poor electrical properties, the *ZT* peak value of SiGe is not very high (1.3 at 1200 K) [10]. Given this, further research into alternative high-temperature thermoelectric material candidates is imperative.

In this work, the thermoelectric properties of alloys from the carbon family (C, Si, Ge, Sn, and Pb) are systematically evaluated. Density functional theory (DFT) [11,12] is applied to investigate the electronic energy band structures of all possible group 14 alloys. Electron–phonon coupling theory is used to determine the electronic relaxation time of the materials. SiSn is found to be a superior thermoelectric candidate compared to SiGe. At present, SiGe alloys are a traditional thermoelectric material [13,14,15,16]. However, Ge is rather rare in the Earth and much more expensive than Sn, and Ge is toxic but Sn is not. Most importantly, SiSn is predicted to have lower lattice thermal conductivity than SiGe, which suggests that SiSn has a greater advantage over SiGe for thermoelectrics [17]. Moreover, the mechanical, optical, thermal, and electrical properties of SiSn are also predicted by many theoretical scientists [18,19]. According to our theoretical predictions, the *ZT* peak value of SiSn can exceed 2.0 at 800 K.

## 2. Computational Methods

In this study, all electrical properties are calculated based on a 12 × 12 × 12 ***k*** mesh within the DFT framework. The Perdew–Burke–Ernzerhof (PBE) with the generalized gradient approximation (GGA) [20] is used for exchange and correlation energy in structural optimization and band structure computations. In the Vienna Ab-initio Simulation Package (VASP) [21,22,23,24], the hybrid Heyd–Scuseria–Ernzerhof (HSE06) functional [25,26] is further utilized to estimate the energy band. The cutoff energy for the wave function is 450 eV. Furthermore, the quasiparticle GW approximation [27], which is implemented in the Quantum ESPRESSO (QE) [28] and Yambo [29,30] packages, is also applied to calculate the electronic energy band. The corresponding cutoff energy is 50 Ry.

Based on electron Boltzmann transport theory, the electron transport parameters involved in the thermoelectric euphoria calculations can be accurately computed. The electrical conductivity and Seebeck coefficient are determined by the transport distribution function $\Xi =\sum_{k}v_{k}v_{k}\tau_{e,k}$, where $v_{k}$ is the group velocity and $\tau_{e,k}$ is the electron relaxation time of the wave vector **k**.

(2) $$\sigma =e^{2}\int \Xi (\varepsilon )\left(-\frac{\partial f_{0}}{\partial \varepsilon }\right)d\varepsilon$$

(3) $$S=\frac{ek_{B}}{\sigma }\int \Xi (\varepsilon )\left(-\frac{\partial f_{0}}{\partial \varepsilon }\right)\frac{\varepsilon -\mu }{k_{B}T}d\varepsilon$$

where $k_{B}$ is the Boltzmann constant, ε is the electronic energy, *μ* is the chemical potential, and $f_{0}$ is the Fermi distribution function.

The electron relaxation time $\tau_{e}$ in the electron transport is an important parameter. Based on Fermi’s golden rule [31,32], the $\tau_{e}$ is given by

(4) $${\left.\tau_{e}^{-1}\right|}_{nk\to mk+q}=\frac{2\pi }{\hbar }{\left|g_{nm}\left(k,q\right)\right|}^{2}\delta \left(\varepsilon_{nk}-\varepsilon_{mk+q}\right)$$

where $g_{nm}\left(k,q\right)$ is the electron–phonon coupling matrix element of electron wave vector **k** and phonon wave vector **q**, and $\varepsilon_{nk}$ is the electron eigenvalue energy of the band *n* and wave vector **k**. In the electron–phonon coupling, the electron–phonon coupling matrix element is defined as

(5) $$g_{mn}(k,q)=\frac{1}{\sqrt{2\omega_{q}}}\langle \psi_{mk+q}\left|\partial_{q}V\right|\psi_{nk}\rangle$$

where $\omega_{q}$ is the phonon frequency of wave vector **q**, $\psi_{nk}$ is the wavefunction of the band *n* and wave vector **k**, and V is the self-consistent potential. Acoustic phonon scattering (APS) and polar optical phonon scattering (POPS) are the two primary scattering mechanisms in electron–phonon coupling, and the electron relaxation time is generally determined by APS. To determine which scattering mechanism dominates in the diamond system, the electron relaxation time of APS and POPS is roughly estimated using a 33 × 33 × 33 **k** mesh. The electron–phonon Wannier (EPW) [31,32] method is used to determine the electron transport properties more accurately. This method must account for all types of phonons (acoustic and optical phonons) in electron scattering, which necessitates a dense electron–phonon mesh in the Brillouin zone, as well as high computing costs [31]. The electron–phonon coupling has been extensively studied and shown to be useful in determining the electrical transport properties of diamond-like structures [33,34,35]. The electron and thermal transport in the electron–phonon coupling are calculated using a 12 × 12 × 12 **k** mesh and 6 × 6 × 6 **q** mesh, respectively. By using the maximally localized Wannier functions interpolation method [36], the electrical and phonon properties of fine meshes of 60 × 60 × 60 **k** mesh and 60 × 60 × 60 **q** mesh can be obtained.

The special quasi-random structure (SQS) [37,38] method is applied to simulate the disordered structure of SiGeSn alloys. Here, 2 × 2 × 2 supercells are constructed by extending the two-atom primitive cell along with the directions of three basis vectors. The objective function of the established randomness of SiGeSn alloys is close to −1.0, indicating that the disordered structures established by SQS may accurately reflect the actual alloy structure. The energy band is also calculated using the HSE06 functional.

The minimum lattice thermal conductivity of SiGeSn alloys can be estimated using Cahill’s model [39], i.e.,

(6) $$\kappa_{\min}={\left(\frac{\pi }{6}\right)}^{1/3}k_{B}\rho^{2/3}\overset{3}{\underset{i=1}{\sum }}v_{i}{\left(\frac{T}{\Theta_{i}}\right)}^{2}\int_{0}^{\Theta_{i}/T}\frac{x^{3}e^{x}}{{\left(e^{x}-1\right)}^{2}}dx$$

Here, ρ is the number density of atoms, $v_{i}$ is the sound velocity of different phonon modes, and Θ is the Debye temperature. This formula is based on the amorphous limit; however, the vibration behavior of atoms in disordered alloys is comparable to that of atoms in amorphous solids; therefore, the model is well suited for predicting disordered SiGeSn alloys.

## 3. Results and Discussion

**Figure 1:**
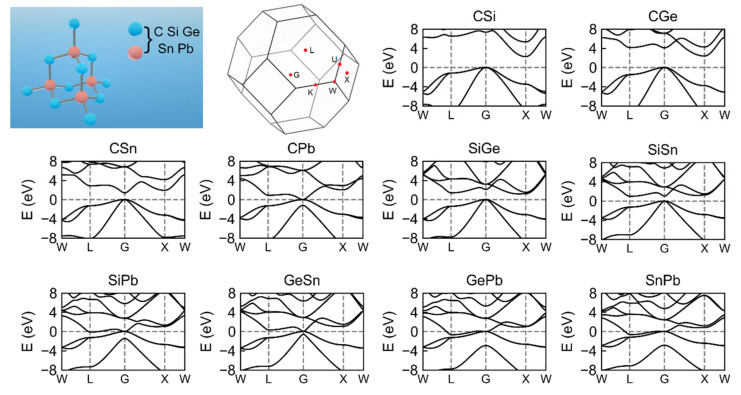
The crystal structure diagram, high symmetry points in the first Brillouin zone, and band structures of group 14 alloys from HSE06 functional.

Alloys containing group 14 elements (C, Si, Ge, Sn, and Pb) have a face-centered cubic structure similar to diamond. Each atom possesses four strong covalent bonds (Figure 1), which guarantees the structure’s rigidity and hardness. As the radius of the atoms in alloys increases, the lattice constants increase correspondingly. Here, the thermoelectric properties of binary alloys are studied firstly, which is used as a basis to screen ternary alloys with better performance. Table 1 presents the optimized lattice constants of binary alloys. The calculated lattice constant of CSi (4.38 Å) is close to the experimental value (4.37 Å) [40]. The table also includes the results of Wang’s LDA computation for comparison [41], and our results are consistent with these data.

The energy band gap *E_g_* plays an important role in predicting thermoelectric performance, since thermoelectric materials need a sufficiently wide band gap to maintain a suitably high electrical conductivity and Seebeck coefficient. For C-based alloys (CSi, CGe, CSn, and CPb), the band energy of the G point decreases as the average molecular mass increases. This modification transforms C-based alloys from indirect semiconductors (CSi and CGe) to direct semiconductors (CSn) to semimetals (CPb). The top of the valence band of these compounds’ energy bands is at the position of the G point. The conduction band bottom of CSi and Cge is situated at high symmetry point X, whereas that of CSn and CPb is positioned at the G point. The PBE and HSE06 calculations predict that the band gaps of CSi are 1.37 and 2.26 eV, respectively, while the experimental measurement is 7.34 eV [43]. Even in the calculation of the HSE06 hybrid functional, the band gap is still underestimated, implying that the actual gap of the material should be considerably larger than our calculated value. Since the calculated gap of CGe is larger than that of CSi, CGe should be an insulator. Among the other group 14 alloys, SiPb, GePb, SnPb, and GeSn are semimetals or metals; thus, further investigation into their thermoelectric properties is superfluous. Therefore, only the three semiconductor materials CSn, SiGe, and SiSn merit further investigation. 

To further screen candidates for n-type thermoelectric materials better than SiGe from these three materials, a type of dimensionless material parameter *β* is introduced to assess the performance [44]. The larger the *β* value, the better the thermoelectric performance. The material parameter *β* can be expressed as

(7) $$\beta =\frac{N_{v}}{3\pi^{2}}{\left(\frac{2m_{d}^{*}k_{B}T}{\hbar^{2}}\right)}^{\frac{3}{2}}\frac{k_{B}^{2}T\tau_{e}}{m_{c}^{*}k_{l}}$$

where $N_{v}$ is the band degeneracy, $m_{d}^{*}$ is the effective mass of density of states, ℏ is the reduced Planck constant, $\tau_{e}$ is the relaxation time of an electron, and $m_{c}^{*}$ is the conductivity effective mass. The $m_{d}^{*}$ and $m_{c}^{*}$ are defined as

(8) $$m_{d}^{*}={\left(m_{x}^{*}m_{y}^{*}m_{z}^{*}\right)}^{1/3}$$

(9) $$\;\frac{1}{m_{c}^{*}}=\frac{1}{3}\left(\frac{1}{m_{x}}+\frac{1}{m_{y}}+\frac{1}{m_{z}}\right)$$

where $m_{i}^{*}$(*i* = *x*, *y,* and *z*) is the electron effective mass in different directions. In general, the APS is dominant in electron transport, and thus the relaxation time $\tau_{e}$ is [45]

(10) $$\tau_{e}=\frac{\pi \hbar^{4}C}{\sqrt{2}E^{2}{\left(m_{d}^{*}k_{B}T\right)}^{3/2}}$$

Here, C is the elastic modulus, and *E* is the deformation potential constant. Then, the *β* is simplified to

(11) $$\beta =\frac{2k_{B}^{2}T\hbar CN_{v}}{3\pi m_{c}^{*}E^{2}k_{l}}.$$

According to the formula, larger band degeneracy and lower conductivity effective mass imply better thermoelectric performance.

**Figure 2:**
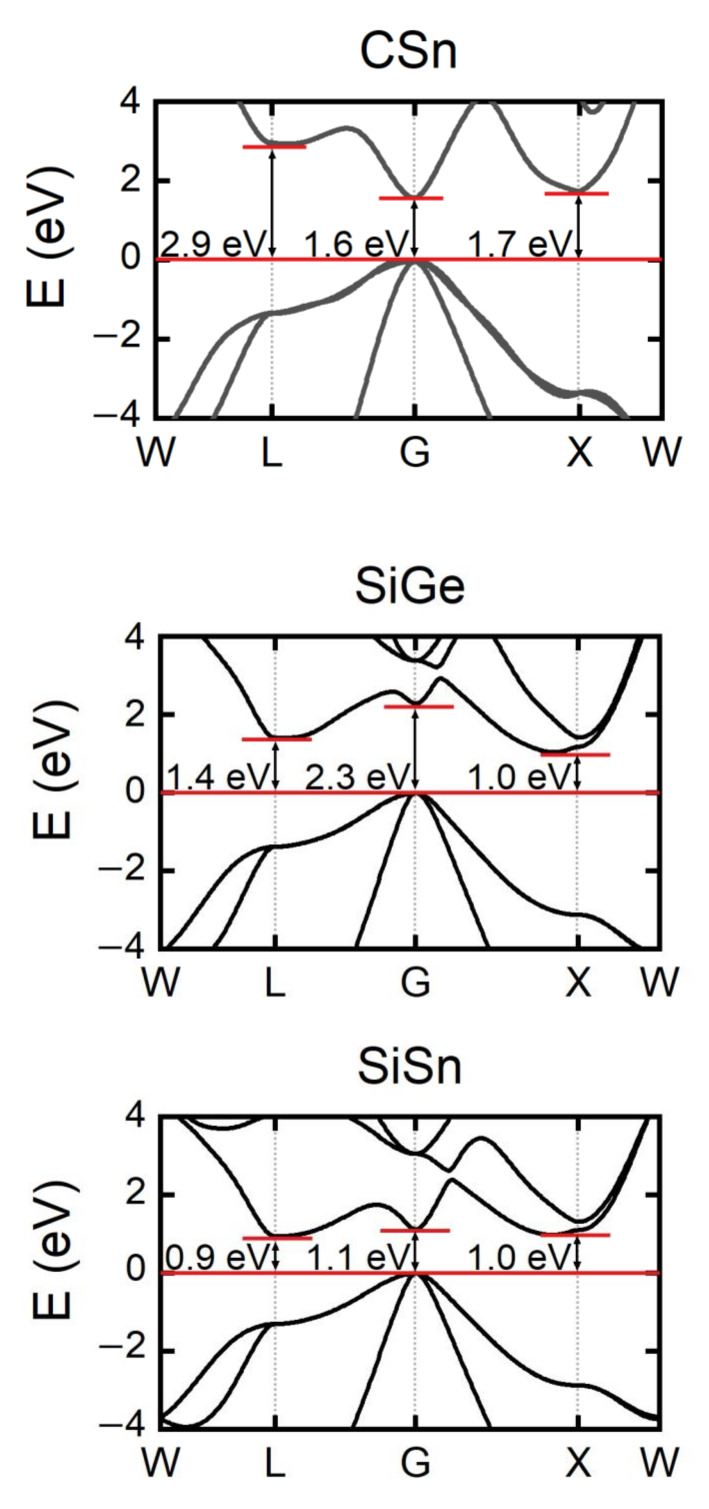
The band structures of CSn, SiGe, and SiSn from GW approximation.

As shown in Figure 2, the band structures of GW are comparable to those of HSE06. The findings of the GW computation are mostly discussed in this section. The gaps between the conduction band energy valleys of G, L, and X and the top of the valence band for CSn are 1.6, 2.9, and 1.7 eV, respectively. The gaps between the conduction band energy valleys of G, L, and X and the top of the valence band in SiGe are 2.3, 1.4, and 1.0 eV, respectively. The gap at the G point is the largest and is 1.15 eV higher than the bottom of the conduction band. The gaps between the conduction band energy valley of G, L, and X and the top of the valence band for SiSn are 1.1, 0.9, and 1.0 eV, respectively. It is worth noting that the difference between the three gaps is small, with the maximum gap difference being no more than 0.2 eV. The degeneracy order of G, L, and X is as follows: L > X > G. Therefore, the conduction band degeneracy order of the three materials is as follows: SiSn > SiGe > CSn.

Table 2 shows the effective masses of CSn, SiGe, and SiSn. Due to the strong isotropy of the conduction bands at the G point, $m_{d}^{*}$ and $m_{c}^{*}$ are found to be equal. The conduction bands of the L and X points are anisotropic, resulting in a discrepancy between $m_{d}^{*}$ and $m_{c}^{*}$. When comparing *β* values, $m_{c}^{*}$ is more significant. It can be seen that $m_{c}^{*}$ is sorted as follows: CSn > SiGe > SiSn. When combined with conduction band degeneracy and conductivity effective mass, it is seen that SiSn has superior thermoelectric properties to SiGe, and therefore SiSn merits further investigation.

**Figure 3:**
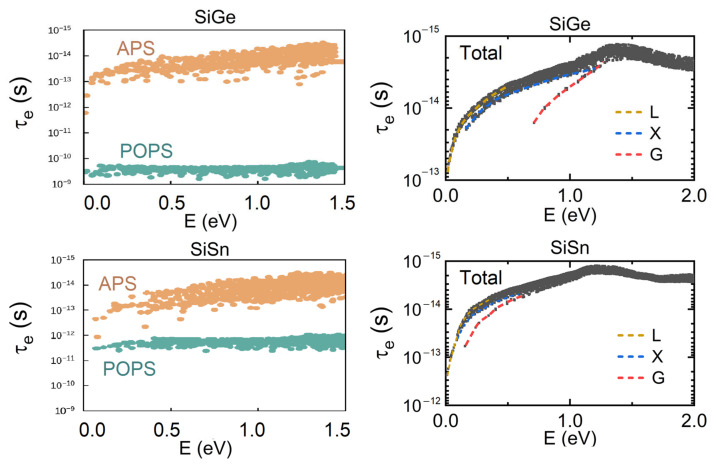
The electronic relaxation time of n-type SiGe and SiSn from electron–phonon coupling at 800 K.

Figure 3 depicts the n-type SiGe and SiSn electron relaxation time from various scattering mechanisms; where the energy is zero, this represents the conduction band’s bottom. At high temperatures, electron–phonon coupling often dominates electron scattering, and thus 800 K is used as a reference. Because the relaxation time of APS is much shorter than that of POPS, APS is stronger than POPS for these two materials. Total relaxation time can be obtained by combining the two scattering mechanisms. Each of the energy valleys is observed to have a distinct relaxation time. The L energy valley has the lowest energy and therefore contributes the most to electron transport. SiSn has a lower electronic effective mass at the G point conduction band energy than SiGe, and thus the relaxation time at the G point increases from 10^−14^ s for SiGe to 10^−13^ s for SiSn. This difference is significant, indicating that SiSn should have an excellent electron transport performance compared to SiGe.

**Figure 4:**
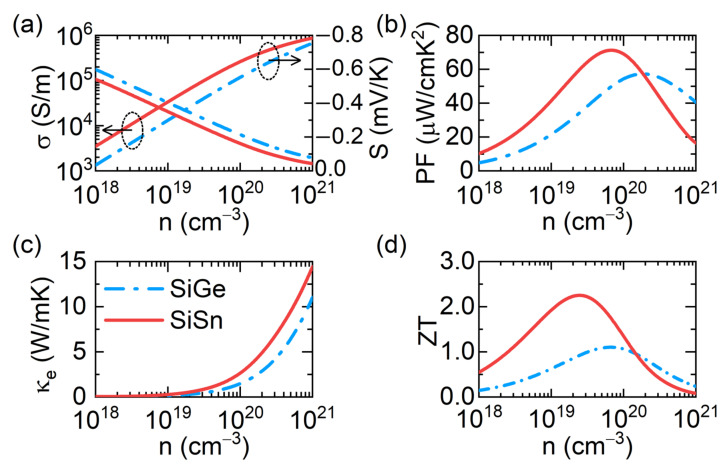
The electrical conductivity *σ* (**a**), Seebeck coefficient *S* (**a**), PF (**b**), electronic thermal conductivity *κ_e_* (**c**), and *ZT* (**d**) of n-type SiGe and SiSn at 800 K.

The electron transport properties of n-type SiGe and SiSn at 800 K at carrier concentrations ranging from 10^18^ to 10^21^ ${\mathrm{cm}}^{-3}$ are shown in Figure 4. Many experiments on the thermoelectric properties of Si_0.8_Ge_0.2_ [46,47,48,49,50,51] have been extensively performed. Because Si_0.8_Ge_0.2_ contains more silicon than Si_0.5_Ge_0.5,_ it has a much wider energy band gap. Comparatively, Si_0.8_Ge_0.2_ has a higher Seebeck coefficient and smaller electrical conductivity. Si_0.8_Ge_0.2_ has a Seebeck coefficient of 0.25 mV/K at 800 K and conductivity of 5 × 10^4^ S/m at an electron carrier concentration of 2.2 × 10^20^ ${\mathrm{cm}}^{-3}$ [10]. Furthermore, at donors of 2.5 × 10^20^ ${\mathrm{cm}}^{-3}$, the Seebeck coefficient of Si_0.7_Ge_0.3_ is 0.21 mV/K [52]. At the 2.2 × 10^20^ ${\mathrm{cm}}^{-3}$ carrier concentration, the two parameters for Si_0.5_Ge_0.5_ are 0.16 mV/K and 2.3 × 10^5^ S/m, respectively. These findings are consistent with our predictions, and our computations are accurate. The PF peak value, on the other hand, is a useful indicator of the electron transport performance. At 800 K, SiGe has a maximum PF of 57 mW/cmK^2^, whereas SiSn has a peak PF of 71 mW/cmK^2^. In contrast, the PF of SiSn is 25% higher than that of SiGe. The impact of multi-energy valleys can enhance the number of conducting channels, resulting in superior electron transport performance.

Thermal conductivity is mainly composed of the contributions from electrons and phonons. According to the Wiedemann–Franz law [53], electronic thermal conductivity is $\kappa_{e}\;=\;L_{0}\sigma T$. However, the relationship between $\kappa_{e}$ and carrier concentration is not linear, indicating that the Lorentz number $L_{0}$ is not constant and varies depending on the carrier concentration. The Lorentz number $L_{0}$ is given by

(12) $$L_{o}={\left(\frac{k_{B}}{e}\right)}^{2}\left\{\frac{\left(r+\frac{7}{2}\right)F_{r+5/2}}{\left(r+\frac{3}{2}\right)F_{r+1/2}}-{\left[\frac{\left(r+\frac{5}{2}\right)F_{r+3/2}}{\left(r+\frac{3}{2}\right)F_{r+1/2}}\right]}^{2}\right\}$$

Here, *r* is the scattering parameter, and the Fermi integral $F_{s}$ is defined as

(13) $$F_{s}=\int_{0}^{\infty }\frac{\varepsilon^{s}/k_{B}T}{e^{\left(\varepsilon -\varepsilon_{F}\right)/k_{B}T}+1}d(\varepsilon /k_{B}T)$$

where $\varepsilon_{F}$ is the Fermi energy. The Lorentz constant is very sensitive to the scattering mechanism. According to the previous analysis, APS dominates the electron transport mechanism in SiGe and SiSn; therefore, *r* is −1/2. The calculated electron thermal conductivity of SiGe and SiSn is illustrated in Figure 4c.

The calculation of the lattice thermal conductivity of group 14 alloys is complex owing to the numerous phonon scattering mechanisms: phonon–phonon, isotope, mass disorder, impurity [54], electron–phonon coupling [34], and nanoparticles [10,55]. There is an alloy disorder (a combination of mass and interatomic local strain field variation), and the effect enhances phonon scattering while significantly reducing lattice thermal conductivity. There are numerous theoretical [17,52,54,56,57,58] and experimental [51,59,60] studies about the lattice thermal conductivity of group IVA alloys. The experimental measurement results show that the lattice thermal conductivity of SiGe at 800 K is approximately 3.8 W/mK [59], while that of nano SiGe is approximately 2.6 W/mK [10]. Furthermore, Khatami and Aksamija computed the thermal transport properties of SiGe and SiSn by using the phonon Boltzmann transport theory [17]. Their calculations fit well with the experiment results, and they claimed that the lattice thermal conductivity of SiSn would be around half of that of SiGe under the same grain scale, and thus the lattice thermal conductivity of SiSn at 800 K is cautiously estimated to be 1.3~1.9 W/mK. Our computed phonon dispersion curves are shown in Figure S1, which suggests that SiSn is as thermodynamically stable as SiGe. Our lattice thermal conductivity of SiGe and SiSn at the amorphous limit at 800 K is 2.6 and 1.4 W/mK, respectively, which is in excellent agreement with the previous theoretical and experimental data.

The *ZT* values of SiGe and SiSn at 800 K can be calculated using Formula (1). The maximum *ZT* value for SiGe is 1.1, which is consistent with the experimental data [10]. This is sufficient to demonstrate the accuracy of our calculations, which are reliable. At the optimum carrier concentration, the lattice thermal conductivity of SiSn is considerably lower, and the *ZT* can reach 2.2 for SiSn. SiSn had a *ZT* value that was approximately 100% more than SiGe. This incredible finding offers new possibilities for group 14 alloys with diamond-like structures, and SiSn may be a potential thermoelectric material to replace SiGe.

**Figure 5:**
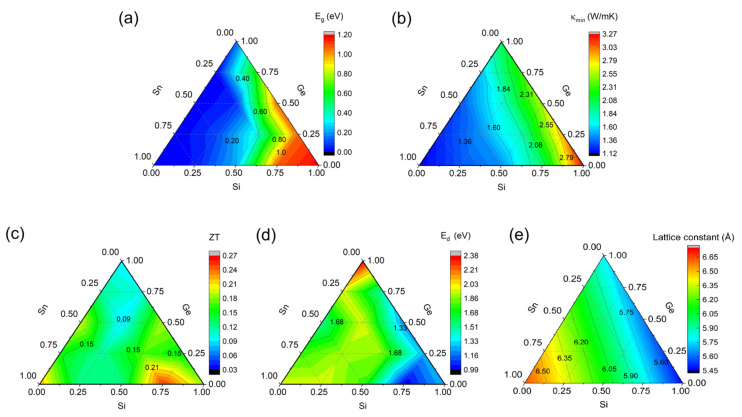
The band gap E_g_ (**a**), lattice thermal conductivity $\kappa_{\min}$ at 800 K (**b**), n-type ZT at 800 K (**c**), band difference $E_{d}$ (**d**), and optimized lattice constant distribution (**e**) of SiGeSn alloys.

However, alloys include not only binary alloys with the same ratios of two compositions, but also binary alloys with different proportions, ternary alloys, and so on. SiGeSn alloys are chosen for a further search for high-performance alloys with additional components based on the appropriate energy gap and relatively low lattice thermal conductivity. Figure 5e depicts the optimal lattice constant distribution of SiGeSn alloys. The lattice constants at different ratios establish a linear relationship, which is in line with Vegard’s law. Figure 5a depicts the band gaps of each alloy based on the calculation of the HSE06 functional, and Figure 5b shows the lattice thermal conductivity at the amorphous limit. The greater the Sn component, the narrower the band gap, and the lower the lattice thermal conductivity. Assuming that the electron relaxation time is 10^−15^ s, the *ZT* of SiGeSn alloys can be determined by constant relaxation time approximation [61]. This relaxation time value is very conservative, particularly in comparison to the results from electron–phonon coupling. Although the *ZT* of alloys is underestimated, it can reflect the relative thermoelectric performance. It has been found that Si_0.75_Sn_0.25_ has superior thermoelectric performance to Si_0.5_Sn_0.5_, which means that SiSn alloys continue to offer unmatched benefits over SiGe alloys.

The gaps between the three conduction band valleys (G, X, and L) and the top of the valence band are $E_{G}$, $E_{X}$, $E_{L}$, respectively. Band difference $E_{d}$ is defined as

(14) $$E_{d}=E_{G}+E_{X}+E_{L}-3\min\{E_{G},E_{X},E_{L}\}$$

The smaller $E_{d}$ represents the three conduction band valleys at the G, X, and L points, which converge to each other more closely. Si_0.75_Sn_0.25_ and Si_0.75_Ge_0.25_ possess strong energy band convergence, as shown in Figure 5d. Considering that Si_0.75_Sn_0.25_ has lower lattice thermal conductivity, it is no wonder that Si_0.75_Sn_0.25_ has superior thermoelectric performance. Therefore, for a material to have good thermoelectric properties, it must have a sufficient band gap in addition to strong band degeneracy and low lattice thermal conductivity.

## 4. Conclusions

In this study, we investigated the differences in the energy band structures of group 14 (C, Si, Ge, Sn, Pb) alloys by using rigorous first-principles calculations. As a traditional thermoelectric material, n-type SiGe benefits from the effect of conduction band energy degeneracy, which also exists in SiSn. Surprisingly, SiSn alloys possess stronger energy band convergence; namely, the energy values of conduction band valleys at the L and X points are very close to the energy at the G point, forming a three-energy-valley convergence effect. The electron transport properties of SiSn are indeed found to be superior to those of SiGe by using Boltzmann transport theory and electron–phonon coupling theory. This finding demonstrates that the multi-energy valley effect can effectively enhance electron transport performance. 

Furthermore, due to the stronger mass–disorder scattering, the lattice thermal conductivity of SiSn is lower than that of SiGe. SiSn exhibits ZT values greater than 2.0 at 800 K, which supports its superior electron transport performance and lower lattice thermal conductivity. The theoretical calculation suggests that SiSn has very high ZT values (up to 2.2 at 800 K), implying that SiSn may be a potential new candidate for thermoelectric materials. A further in-depth investigation reveals that the best thermoelectric component of SiSn alloys is Si_0.75_Sn_0.25_, which is enhanced by strong band degeneracy and low lattice thermal conductivity.

## Figures and Tables

**Table 1:** The lattice parameters of group 14 alloys, and the band gaps *E_g_* from PBE and HSE06 calculation. Lattice parameters from another ab initio calculation [41] (Reprinted with permission from Ref. [41]. Copyright 2002, American Physical Society) and experimental data [41,42] are listed for comparison.

	*a* from QE (Å)	*a* from VASP (Å)	*a* from [41] (Å)	*a* from Exp. (Å)	*E_g_* of PBE (eV)	*E_g_* of HSE06 (eV)
CSi	4.38	4.38	4.314	4.360 [41]	1.37	2.26
CGe	4.63	4.63	4.500		1.62	2.40
CSn	5.09	5.10	4.961		0.64	1.38
CPb	5.27	5.38	5.139		0.00	0.00
SiGe	5.61	5.62	5.472	5.537 [42]	0.61	1.17
SiSn	5.99	6.08	5.914		0.40	0.97
SiPb	6.29	6.32	6.047		0.00	0.00
GeSn	6.22	6.22	6.004		0.00	0.14
GePb	6.42	6.46	6.154		0.00	0.00
SnPb	6.80	6.86	6.539		0.00	0.00

**Table 2:** The effective mass of CSn, SiGe, and SiSn.

	$m_{d}^{*}(m_{e})$			$m_{c}^{*}(m_{e})$		
	G	L	X	G	L	X
CSn	0.08	0.40	0.37	0.08	0.26	0.32
SiGe	0.08	0.18	0.32	0.08	0.14	0.28
SiSn	0.04	0.16	0.27	0.04	0.13	0.26

## Supplementary Materials

The following supporting information can be downloaded at: www.mdpi.com/article/10.3390/ma15124107/s1, Figure S1: Phonon dispersion curves of SiSn and SiGe.
