# Supplementary material for: First-Principles Study of Silicon–Tin Alloys as a High-Temperature Thermoelectric Material
Source: Materials (Basel). 2022 Jun 9;15(12):4107. doi: 10.3390/ma15124107 (PMC9229319; doi:10.3390/ma15124107)
Supplement: Supplementary file 1 [file materials-15-04107-s001.zip › materials-1736390-supplementary.pdf]

Supporting Information

First-Principles Study of Silicon-Tin Alloys as a High- Temperature Thermoelectric Material

Shan Huang, Suiting Ning and Rui Xiong*

Key Laboratory of Artificial Micro- and Nano-Structures of Ministry of Education and School of Physics and Technology, Wuhan University, Wuhan 430072, China; shanhuang@whu.edu.cn (H.S.); stning@whu.edu.cn (S.N.)

* Correspondence: xiongrui@whu.edu.cn (X.R.)

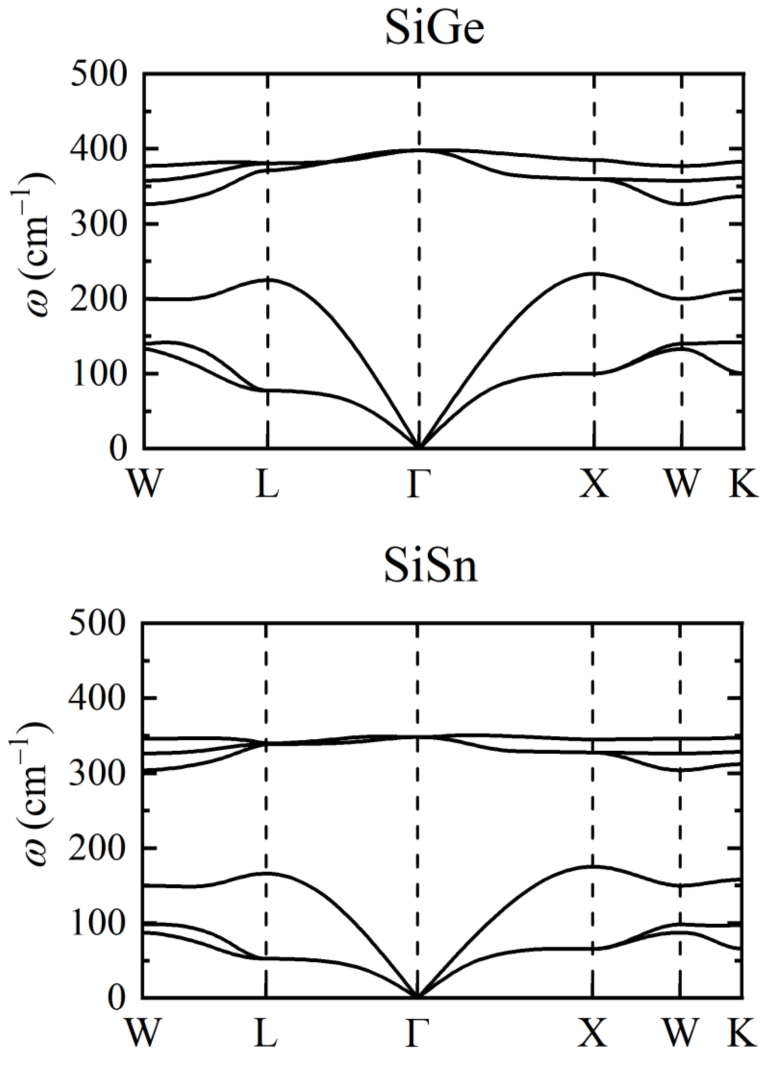

Figure S1. The phonon dispersion curves of SiGe and SiSn.
